# Role of Plasmapheresis and Extracorporeal Membrane Oxygenation in the Treatment of Leptospirosis Complicated with Pulmonary Hemorrhages

**DOI:** 10.1155/2018/4520185

**Published:** 2018-12-02

**Authors:** C. L. Fonseka, S. Lekamwasam

**Affiliations:** Department of Internal Medicine, Faculty of Medicine, University of Ruhuna, Sri Lanka

## Abstract

**Introduction:**

Leptospirosis is an emerging infectious disease associated with multiorgan involvement and significant morbidity and mortality. Although pulmonary hemorrhage due to leptospirosis has a high fatality, specific treatment options are limited and their efficacy is not adequately proven. We opted to find out the current evidence on plasmapheresis and extracorporeal membrane oxygenation (ECMO) in pulmonary hemorrhages due to leptospirosis.

**Methods:**

The first search was conducted in PubMed, OVID, Google Scholar, and Cochrane clinical trial registry using keywords “leptospirosis” OR “Leptospira” OR “Weil's disease” AND “plasmapheresis” OR “plasma exchange” AND “pulmonary hemorrhage” OR “alveolar hemorrhage” OR “lung hemorrhage” and the second search was done using keyword “leptospirosis” OR “Leptospira” OR “Weil's disease” AND “ECMO” OR “Extracorporeal membrane oxygenation.” The searches were not limited by study design or the date of publication. Only articles written in English were reviewed. Although we intended to include only clinical trials, it was decided later to include other information such as case reports and case series which addressed these treatment modalities. Two authors selected articles independently in a blinded manner using a set of inclusion and exclusion criteria and discrepancies were solved after discussions.

**Results:**

The information found was very limited. This included one clinical trial which showed a significant survival benefit with plasmapheresis but the study design had many limitations. Two case reports described the benefit of plasmapheresis in severe leptospirosis with pulmonary hemorrhages. There were eight case reports where ECMO was performed and out of all only one patient has died. One retrospective study on patients with severe leptospirosis mentioned that four out of five patients with pulmonary hemorrhages survived after being treated with ECMO.

**Conclusions:**

Current evidence is insufficient to recommend the routine use of plasmapheresis or ECMO for patients presenting with pulmonary hemorrhages due to leptospirosis. ECMO may be a promising mode of treatment in acute respiratory failure in leptospirosis related pulmonary hemorrhages. These treatment modalities, however, can be applied based on the availability of resources and expertise at the discretion of the clinician in charge, considering patient related factors such as cardiovascular stability and derangement of coagulation profile. Clinical trials conducted adhering to standard procedures are urgently required to establish the efficacy of these treatment modalities.

## 1. Background

Leptospirosis is an emerging infectious disease with increasing incidence in both developing and developed countries [1]. It has a wide geographical distribution and is observed commonly in tropical, subtropical, and temperate zones, reaching endemic proportions in South East Asian region [2]. Globally, around 10,000 severe cases of leptospirosis require hospitalization every year [1]. The major burden attributed to leptospirosis has been the severe life-threatening manifestations. Leptospirosis has a mean case fatality ratio of 6.85%, with the highest risk for death among males of 50–59 years of age [3]. Of the disease related complications, pulmonary hemorrhages are the major cause of mortality, accounting for 30-60% of deaths [4–8]. Edilane L* et al*. have reported an increasing detection rate of pulmonary hemorrhages in Brazil in the recent past [9].

Antibiotics are the mainstay of treatment in suspected or confirmed leptospirosis but the efficacy of different antibiotics is uncertain due to the scarcity of clinical trials [10]. In pulmonary involvement where the highest mortality is seen, there is sparse evidence for an effective treatment modality, currently. Although high-dose intravenous glucocorticoids are used in pulmonary involvement, the evidence is weak and is limited to case reports and case series [11]. Clinical trials [12, 13] have shown that glucocorticoids are ineffective and may increase the risk of nosocomial infections. Intravenous cyclophosphamide has been found to be effective in a single nonrandomized trial conducted in India, but the methodological flaws seen in this study restrict the application of the results in patient care [14].

There is a growing interest in plasmapheresis and ECMO as potential treatment modalities for leptospirosis with lung involvement. These treatment modalities require additional resources such as expertise and infrastructure; hence, they are not widely used. Plasmapheresis and ECMO, however, have not been included in the national patient management guidelines in countries where the condition is prevalent. We intended to explore the effectiveness of plasmapheresis or ECMO in a systematic manner using the current evidence.

## 2. Methods

**Figure 1:**
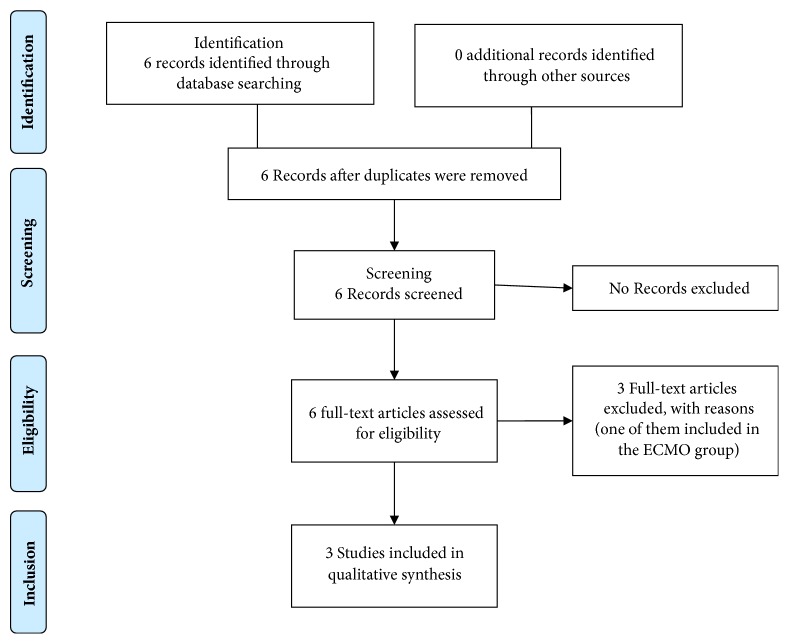
Plasma exchange (last search September 30, 2018).

**Figure 2:**
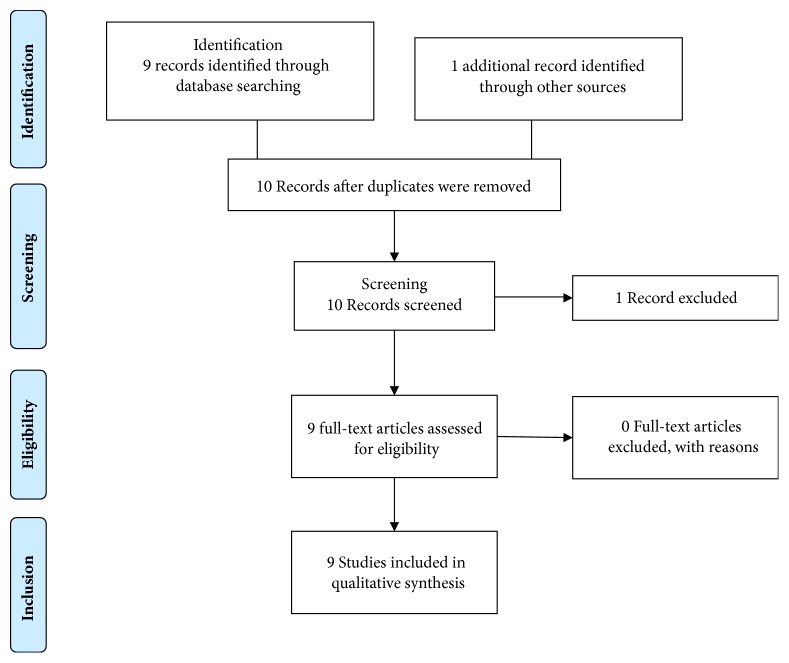
Extracorporeal membrane oxygenation (last search September 30, 2018).

The review was done adhering to the PRISMA protocol. The first search was conducted in PubMed using keywords “leptospirosis” OR “Leptospira” OR “Weil's disease” AND “plasmapheresis” OR “plasma exchange” AND “pulmonary haemorrhage” OR “alveolar haemorrhage” OR “lung haemorrhage” and the second search was done with keywords “leptospirosis” OR “Leptospira” OR “Weil's disease” AND “ECMO” OR “Extracorporeal membrane oxygenation.” Last search was done on September 20, 2018 (Figures 1 and 2). The searches were not limited by study design or the date of publication. Only articles written in English were reviewed. One article that did not have an English translation was excluded. A similar search was conducted on OVID, Google Scholar, and the Cochrane clinical trial registry. Although our initial intention was to include only clinical trials, we decided to include other forms of information such as case reports and case series, which addressed plasmapheresis or ECMO in leptospirosis. Other types of publications such as reviews and comments were excluded. Reference lists of included articles were also perused. No additional eligible articles were found. Two authors selected articles independently in a blinded manner using a set of inclusion and exclusion criteria and discrepancies were sorted out after discussion.

## 3. Results

### 3.1. Evidence on Plasmapheresis for Patients with Leptospirosis Pulmonary Hemorrhage (Table 1)

We found two case reports [15, 16] and one clinical trial [17], where benefits of plasmapheresis in severe leptospirosis with pulmonary hemorrhages have been described. Chen et al. [15] described a patient with leptospirosis and coinfection with typhus presenting with diffuse alveolar hemorrhage and acute kidney injury (AKI) and recovered after 8 cycles of plasma exchange and high-dose steroids. Despite recovery from pulmonary hemorrhage, this patient had a complicated course of illness with active gastrointestinal bleeding and necessitated 6 weeks of hospitalization. Dursan B et al. [16] have described a patient with severe alveolar hemorrhages and AKI following leptospirosis and recovered after 9 cycles of plasmapheresis. Trivedi et al. [17] described a nonrandomized trial in which first 30 patients received conventional therapy with antibiotics and steroids and next consecutive 114 patients received plasmapheresis and cyclophosphamide. This study showed a high mortality benefit in the plasmapheresis group when compared with the conventional treatment (61.4% vs 16.6%). The study only recruited patients with mild pulmonary hemorrhages with an acute lung injury score less than 25 and there were limitations in the study design.

### 3.2. Evidence on Extracorporeal Membrane Oxygenation for Patients with Leptospirosis Pulmonary Hemorrhage (Table 2)

There were eight case reports and one retrospective study where ECMO is used in patient with leptospirosis with pulmonary hemorrhage [25–33]. Seven case reports [25–31] showed benefits of ECMO in patients with leptospirosis while one report [32] described treatment failure despite using variety of advanced invasive treatment measures. Also, in a retrospective study of 134 leptospirosis cases treated in ICU, five have undergone ECMO, out of which four have survived [33]. In individual case reports, Pardinas et al. [25] described a patient presenting with massive hemoptysis and persistently low oxygenation despite ventilation, recovering after 18 days of venovenous ECMO (vv-ECMO). This patient has received concurrent aminocaproic acid infusion to reduce pulmonary bleeding. Liao et al. [26] describe a patient with severe leptospirosis complicated by massive pulmonary hemorrhage with fresh bleeding from endotracheal tube leading to refractory hypoxemia and hypercapnia despite ventilation recovering after 6 days of vv-ECMO. A 50-year-old patient who had leptospiremic septic shock with acute respiratory failure underwent vv-ECMO where higher blood flow rates were used with a lower activated partial thromboplastin time (40-50sec) due to the bleeding in the initial three days. He recovered after 11 days of ECMO despite being complicated with acute kidney injury (AKI) requiring renal replacement therapy and cardiomyopathy with an ejection fraction of 30% [27]. A patient who had a sudden cardiac arrest due to severe hypoxemia subsequent to lung haemorrhage recovered after 183 hours of ECMO [28]. This patient required molecular adsorption recycling system (MARS) in order to reduce hyperbilirubinemia. Cantwell et al. describe an obese 39-year-old patient with lung hemorrhage requiring vv-ECMO with a second membrane oxygenator to improve oxygenation to maintain enough membrane surface and flow due to obesity [29]. Another traveller from Laos recovered after being on ECMO for 9 days [30].

Except in one instance where venoarterial ECMO was used [31], all have used vv-ECMO. There was one report where a patient presenting with established multiorgan failure underwent ventilation, plasmapheresis, vv-ECMO, and continuous renal replacement therapy (CRRT) with extracorporeal cytokine absorbent therapy but, despite all measurements, the patient succumbed [32].

### 3.3. Evidence of Plasmapheresis for Leptospirosis Where Pulmonary Hemorrhage Was Not Either Present or Clearly Mentioned (Table 1)

In some instances, plasmapheresis has been used in situations where pulmonary hemorrhage was not either present or clearly mentioned and they too failed to show a clear treatment benefit. Landini et al. summarized 6 patients with hyperbilirubinemia, AKI, and no-specified hemorrhagic manifestations treated with plasmapheresis and noticed an improvement of hepatorenal function and bleeding [18]. Another patient with AKI and liver and cardiac involvement but without pulmonary involvement has been treated with plasmapheresis combined with continuous renal replacement therapy and high-volume hemofiltration but subsequently developed CMV colitis and resistant bacteremia and required prolonged hospital stay and was released from hospital after 70 days [19]. Another patient with multiorgan involvement (cardiac, renal, and hepatic involvement) and hemoptysis recovered following plasma exchange and systemic steroid therapy [20]. There were two case reports where plasma exchange was performed to reduce the toxic effects of hyperbilirubinemia with the intention of reducing its toxic effects on tissues including kidneys [21, 22]. In both these instances, there was a marked reduction of bilirubin levels by plasma exchange and both these patients had diffuse pulmonary infiltrates in chest radiography and they were not clearly mentioned as pulmonary hemorrhages. There is a case report and a case series where multiorgan involvement was present and plasma exchange was used with continuous venovenous hemofiltration (CVVHF) to assist recovery [23, 24].

## 4. Discussion

In this systematic review, we found no strong evidence to support the routine use of plasmapheresis or plasma exchange in leptospirosis complicated with pulmonary hemorrhage. Current evidence is limited to several case reports and a solitary no-randomized clinical trial. Case reports have inherited publication bias, since treatment failures are unlikely to be reported. The only clinical trial on plasmapheresis is nonrandomized, used patients with mild lung involvement, and used cyclophosphamide as an adjuvant therapy. Furthermore, as the two groups were nonparallel, the type of care provided to them could have been different. Also, the mortality benefits in the intervention group are surprisingly more significant than in the control group. It can be argued that the experience gathered in treating controls first may have helped to provide an improved care for the treatment group subsequently.

The frameworks for using ECMO or plasmapheresis are fundamentally different. Plasmapheresis could be considered a treatment targeting pathogenesis of the disease which may help to remove offending antibodies and immune complexes. But it may be hazardous in inducing dilutional coagulopathy and there is a possibility that protective coagulation factors can be removed during the process. Also, there are concerns that exposure to blood products can lead to critical hemodynamic compromise by giving rise to anaphylaxis. On the other hand, patients with very severe diseases may succumb as the effects of plasmapheresis may not occur immediately as they do not correct the hypoxemia due to the acute respiratory failure. Conversely, ECMO is a symptomatic treatment of respiratory failure, which corrects the persistent hypoxemia, which can lead to multiorgan dysfunction. Its use is more recent than for plasmapheresis and tends to increase (4 articles in 2017), gaining a significant interest in leptospirosis associated pulmonary hemorrhage. In severe cases of lung hemorrhage, experts prefer using ECMO due to the mentioned reasons, especially when the lung involvement is isolated or predominant. Interestingly, although these techniques were previously reserved for developed countries, now their use is spreading in some developing countries (Southeast Asia), especially endemic to leptospirosis.

Another symptomatic treatment was aminocaproic acid [34, 35] to reduce bleeding from lungs and inhaled nitric oxide [36] which has been known to increase pulmonary blood flow to areas of normal ventilation and DDAVP [13, 37]. Desmopressin is known to trigger the release of endothelial haemostatic factors, shortens prolonged bleeding times, enhances platelet adhesiveness, and induces von Willebrand factor secretion by activating endothelial cell V2 receptors. Also, desmopressin has also been proven to be effective in the bleeding associated with hepatic and renal failure [13]. Although in a case series of 6 patients cessation of bleeding was demonstrated with DDAVP [37], a randomized control study of DDAVP disproves this by showing no significant difference compared with the control or steroid treatment groups [13]. Surprisingly, we could not find evidence on using tranexamic acid in leptospirosis pulmonary hemorrhages.

The current National guidelines on leptospirosis in India and Sri Lanka only recommend antibiotics, high-dose corticosteroids, and respiratory support when the disease is complicated with pulmonary hemorrhage. The latest Indian guidelines developed in 2015 have not included plasmapheresis as a treatment option, although the results of the clinical trial by Trivedi et al. were available by then. This is understandable as the treatment benefit shown in this study has not been confirmed by other workers.

We found four case reports [38–41] to support the recommendations made by the national guidelines of Sri Lanka and India [42, 43]. These patients with severe pulmonary hemorrhages were only treated with high-dose corticosteroids and respiratory support and they have recovered fully. In some instances patients have recovered with respiratory support without corticosteroids [44–48].

Lack of evidence, however, is not synonymous with lack of efficacy and it calls for methodologically sound well-conducted clinical trials. Leptospirosis is prevalent in tropical, subtropical, and especially South Asian regions, where clinical trials are infrequent. Financial constraints and lack of resources do not provide an environment conducive for clinical trials in this region. Despite these limitations, urgent attention should be paid to this deadly disease and more clinical trials should be conducted to minimize the high mortality currently seen.

## 5. Conclusion

Current available evidence is insufficient to recommend the routine use of ECMO, plasmapheresis, or plasma exchange for patients presenting with pulmonary hemorrhages due to leptospirosis. These advanced modes of treatment, however, can be applied based on the availability of resources and expertise locally, at the discretion of the clinician in charge, considering each patient individually.

## Figures and Tables

**Table 1:** Summary table for leptospirosis patients treated with plasmapheresis.

**PEX for patients with leptospirosis related pulmonary hemorrhages**					
**Reference**	**Level of evidence**	**Complications of disease**	**Treatment used**	**Other complications**	**Outcome**

Chen Y et al [15]	Case report - coinfection with scrub typhus	Pulmonary hemorrhage, acute renal failure	8 cycles PEX and corticosteroids	Active upper gastrointestinal bleeding	Discharged after 6 weeks of hospitalization

Dursun B et al [16]	Case report	Pulmonary hemorrhage, renal failure	9 PEX, corticosteroids		Recovered

Trivedi SV et al [17]	Nonrandomized nonparallel clinical trial, 2 groups sequentially recruited	Only mild cases (ALI score <2.5) were included	PEX		Control group - 5/30 patients (16.6%) survived Treatment group - PEX & CPP 70/114 patients (61.4%) survived

**PEX when pulmonary hemorrhage was not present or not clearly mentioned**					

Landini et al [18]	Case series of 6 patients	Hyperbilirubinemia and hemorrhagic manifestations (not specified)	PEX		Improvement of hepatorenal function, hemorrhagic state and coma grade

Bourquin V et al [19]	Case report	Multiorgan dysfunction (acute kidney injury, liver failure, myocarditis, thrombocytopenia)	PEX, CRRT, high-volume hemofiltration (HVHF)	Penicillin-resistant enterococcus bacteremia, dry necrosis of both extremities, transient pacing, several respiratory arrests, severe CMV colitis requiring sigmoidectomy and ganciclovir	ICU 45 days and discharged after 70 days

Taylor et al [20]	Case report	Multiorgan dysfunction (hemoptysis with acute respiratory failure, marked hyperbilirubinemia with fulminant liver failure, AKI, AF, shock)	2 PEX, CRRT, corticosteroids		Recovered. CRRT stopped after 9 days

Tse KC et al [21]	Case report	Severe conjugated hyperbilirubinemia with liver failure, acute renal failure, CXR - diffuse bilateral pulmonary infiltrates	PEX		Recovered

Cerdas-Quesada C et al [22]	Case report	Hyperbilirubinemia, liver failure, acute kidney injury, CXR - diffuse bilateral pulmonary infiltrates	5 PEX		Recovered

Yesilbas O et al [23]	Case report	Cardiac arrest, pericardial tamponade, renal failure, macrophage activation syndrome, later suffered prolonged jaundice and sclerosing cholangitis	PEX, continuous venovenous hemofiltration (CVVHF)		Recovered, transferred to ward after 62 days in ICU

Siriwanij T et al [24]	Case series	10 patients' lung crepitations, 2 patients had hemoptysis, hyperbilirubinemia, transaminitis, renal failure	PEX or continuous venovenous hemofiltration (CVVH)		All recovered

*∗*PEX: plasma exchange, ALI: acute ling injury, CRRT: continuous renal replacement therapy, ICU: intensive care unit.

**Table 2:** Summary table for leptospirosis pulmonary hemorrhage (PH) patients treated with ECMO.

**Author**	**Level of evidence**	**Reason to initiate ECMO**	**Respiratory settings recorded before ECMO**	**Advanced treatment modalities**	**Day of PH**	**Complications**	**Outcome**
Pardinas M et al [25]	Case report	Massive hemoptysis and acute hypoxemic respiratory failure (after 36 h of arrival)	Pao2/Fio2 ratio (P/F) <30 mm Hg and plateau pressures >40 cm/H2O, SpO2 74-80% (ACT –160-180 seconds due to persistent hemoptysis)	vv-ECMO (13 days), Aminocaproic acid infusion	D12	Episodic hypotension, AKI on RRT, multiorgan failure	Discharged after 40 days

Liao CY et al [26]	Case report	Refractory acute respiratory failure, severe hypercapnia, continuous bleeding from ET	Pao2/Fio2 ratio (P/F) – 163, pO2 of 65.5 mmHg and pCO2 of 78.1 mmHg and FiO2 of 40%	Venous ECMO (6 days)	D3	No RRT (creatinine 1.6mg/dl), shock	Discharged after 10 days

Umei N et al [27]	Case report	Pulmonary hemorrhage	*F*iO2 – 100%, paO2 - 70.4mmHg, paCO2 -28.3mmHg, PEEP - 10 cm H_2_O	vv-ECMO (11 days)	D5	Septic shock, AKI on RRT, myocarditis	Recovered. Extubated on day 13

Arokianathan D et al [28]	Case report	Pulmonary hemorrhage with progressively decreasing oxygen saturations, 300 ml of fresh blood from endotracheal tube	FiO2 100%, paO2- 7.7kPa, pCO2 - 5.1kPa	vv-ECMO (183 hrs), molecular adsorption recycling system (MARS) for hyperbilirubinemia	D5	AKI, hyperbilirubinemia, cardiac arrest	Recovery

Cantwell T et al [29]	Case report	Pulmonary hemorrhage	PaO2/FiO2 - 89, Murray score 3	vv-ECMO (8 days), high-volume hemofiltration (HVHF), high flow with 2 oxygenators (as the patient is obese)		AKI, septic shock, ARDS, myocarditis	Discharged on day 28

Hery G et al [30]	Case report	Pulmonary hemorrhage with massive hemoptysis	PaO2: FiO2 ratio – 34, FiO2 100%, and PEEP of 10 cm H_2_O.	vv-ECMO (9 days)		Shock, disseminated intravascular coagulation, AKI, lactic acidosis	Discharged after 20 days

Kahn MJ et al [31]	Case report	Pulmonary hemorrhage with progressive hypoxia		Venoarterial ECMO (60 hrs)	D3	Septic shock, myocarditis, atrial fibrillation, AKI on RRT	Discharged on day 26

Ludwig et al [32]	Case report	Pulmonary hemorrhage	pO2 51.8 mmHg, pCO2 60.8mmHg, SpO2 60% on air	vv-ECMO, PEX, CRRT, extracorporeal cytokine absorbent therapy	D1	AKI on RRT, septic shock, ARDS intravascular hemolysis (TTP DIC excluded)	Died 29 hrs after initial symptoms (17 hrs after admission)

Delmas B et al [33]	Retrospective study of 134 ICU leptospirosis admissions		Median Pao2/Fio2 ratio - 155 (85–211) for the 14 patients (10%) undergoing ventilation			Overall mortality rate was 6%, mortality in moderate-to-severe ARDS subgroup was 25%, four patients died from refractory ARDS (one with therapeutic limitations), three from multiple organ failure, and one from nosocomial septic shock	Five patients who underwent ECMO for refractory ARDS, 80% (4 patients) survived

*∗*ECMO: extracorporeal membrane oxygenation, ACT: activated clotting time, AKI: acute kidney injury, RRT: renal replacement therapy, PEX: plasma exchange, CRRT: continuous renal replacement therapy, ARDS: acute respiratory distress syndrome, ICU: intensive care unit.

## Data Availability

All details are included in this published article and are available from included studies which are fully referenced.
